# The Role of Epigenetic in Dental and Oral Regenerative Medicine by Different Types of Dental Stem Cells: A Comprehensive Overview

**DOI:** 10.1155/2022/5304860

**Published:** 2022-06-09

**Authors:** Ahmed Hussain, Hamid Tebyaniyan, Danial Khayatan

**Affiliations:** ^1^College of Dentistry, University of Saskatchewan, Saskatoon, SK, Canada S7N 5E4; ^2^Science and Research Branch, Islamic Azad University, Tehran, Iran; ^3^Faculty of Pharmacy, Tehran Medical Sciences, Islamic Azad University, Tehran, Iran

## Abstract

Postnatal teeth, wisdom teeth, and exfoliated deciduous teeth can be harvested for dental stem cell (DSC) researches. These mesenchymal stem cells (MSCs) can differentiate and also consider as promising candidates for dental and oral regeneration. Thus, the development of DSC therapies can be considered a suitable but challenging target for tissue regeneration. Epigenetics describes changes in gene expression rather than changes in DNA and broadly happens in bone homeostasis, embryogenesis, stem cell fate, and disease development. The epigenetic regulation of gene expression and the regulation of cell fate is mainly governed by deoxyribonucleic acid (DNA) methylation, histone modification, and noncoding RNAs (ncRNAs). Tissue engineering utilizes DSCs as a target. Tissue engineering therapies are based on the multipotent regenerative potential of DSCs. It is believed that epigenetic factors are essential for maintaining the multipotency of DSCs. A wide range of host and environmental factors influence stem cell differentiation and differentiation commitment, of which epigenetic regulation is critical. Several lines of evidence have shown that epigenetic modification of DNA and DNA-correlated histones are necessary for determining cells' phenotypes and regulating stem cells' pluripotency and renewal capacity. It is increasingly recognized that nuclear enzyme activities, such as histone deacetylases, can be used pharmacologically to induce stem cell differentiation and dedifferentiation. In this review, the role of epigenetic in dental and oral regenerative medicine by different types of dental stem cells is discussed in two new and promising areas of medical and biological researches in recent studies (2010-2022).

## 1. Introduction

The epigenetic chromatin state will change without deoxyribonucleic acid (DNA) sequence alterations, leading to gene regulation. This occurs when a signal is received from an initiator, such as noncoding RNAs (ncRNAs), depending on the environmental changes around the cell. An initiator determines which regions of a chromosome need to be affected, thus changing gene expression. Histone modifications and DNA methylation, epigenetic maintainers, allow chromatin to remain markedly epigenetically altered [1]. It is known that stem cells are crucial to the regeneration of damaged tissues, and they come from the embryonic stem cells or the postnatal origin (adult stem cells). The epigenetic state of embryonic stem cells permits self-renewal or differentiation into any pluripotent cell type, unlike adult stem cells, which have more restricted lineage potential (multipotent) [2]. The dental pulp or periodontium, primarily a source of dental stem cells (DSCs), functions as a source of mesenchymal stem cells (MSCs). DSCs positively expressed markers related to MSCs in vitro containing cluster of differentiation (CD)146, 105, 90, 73, 59, 44, 29, 13, and STRO-1. Contrarily, some hematopoietic markers (CD45, 34, 31, and 14) were negatively expressed [3]. In many tissues, including the dental pulp, the renewal of stem cells depends on epigenetic mechanisms to histone proteins. Epigenetics, or the modification of gene expression by environmental factors, is unaffected by any DNA sequence changes [2]. DNA methylation, histone modifications, ribonucleic acid (RNA) modifications, and ncRNAs are generally responsible for epigenetic modifications. The enzyme DNA methyltransferases (DNMTs) are responsible for DNA methylation, one of the well-explored epigenetic modifications. As a result of DNMTs, the 5-methylcytosine existing in CpG islands can be methylated into DNMTs. It is also possible to reverse DNA methylation using enzymes from the ten-eleven translocation (TET) family [3]. DNA methylation regulates the expression of phosphatase and tensin homolog (PTEN), which contributes to tumor progression and resistance to chemotherapy. It was demonstrated that overmethylation of the PTEN promoter inhibits PTEN expression in tamoxifen-resistant breast carcinoma cells and activates Protein Kinase B (AKT) and 5-Azacytidine (5-Aza) methylation of the PTEN promoter and making cells more susceptible to tamoxifen's cytotoxicity. The study demonstrated that although PTEN is essential for modulating auditory progenitors in mice and hematopoietic stem cells in zebrafish, the regulation of PTEN expression by DNA methylation could serve different purposes, as a functional part to manage lineage tumorigenesis and commitment in human adult stem cells. In comparison with adipose MSCs, placental MSCs, hematopoietic stem cells, skin fibroblasts, and osteoblasts, PTEN is not a molecule or functional marker for alveolar bone marrow-derived mesenchymal stem cells (BMSCs). Studying dental MSC characteristics, including the meager tumorigenic potential and individual cell fate of dental MSCs, is essential for developing new functional signatures between dental pulp stem cells (DPSCs) and BMSCs [4]. Furthermore, the Xi and Chen findings exhibited that PTEN is decreased in cultures of human osteosarcoma cells in comparison with osteoblasts [5]. Gong et al. showed that PTEN is less expressed in osteosarcomas than in adjacent tissues in human cell lines [6]. Furthermore, the Freeman et al. experiment briefly stated that the PTEN loss was expected in osteosarcoma [7].

Chromatin is reconstituted or relaxed by histone methyltransferases. Histone H3 is the most widespread histone modification. Repressive Histone H3 lysine 9 (H3K9) and Histone H3 lysine 27 (H3K27) precisely and dynamically regulate the transcriptional regulation of target genes. Also, Histone H3 lysine 79 (H3K79), Histone H3 lysine 4 (H3K4), and Histone H3 lysine 36 (H3K36) are correlated with activation of transcription. A variety of internal modifications of messenger RNA (mRNA), such as N^6^-methyladenosine (m6A), N^1^-methyladenosine (m1A), 5-methylcytosine, and 5-hydroxymethylcytosine, are crucial to mRNA stability. Methyltransferases catalyze the transformation of m6A from one state to another, while demethylases reverse it [3]. DNA methylation and posttranslational modifications are the most studied epigenetic modifications, particularly acetylation, methylation, and phosphorylation of histone proteins. Gene expression changes caused by epigenetic modifications can be targeted by drugs that inhibit enzymes, which can either inherit or accumulate during life [2]. Throughout development, the capacity of cells to specialize and differentiate increases. Stem cells in skeletal muscle, bone marrow, and fat use this process to decide whether they should undergo self-renewal or transition into new cells. Hence, adult stem cells can preserve the homeostasis of tissues by repairing and self-renewal or replacing damaged tissues through differentiation. In recent years, scientists have become increasingly interested in the possibility of replacing damaged cells within an organism. These cell populations are currently of particular interest to researchers trying to understand their regenerative ability and the potential use of these cells for immunotherapy or to treat various diseases. Although it has not been determined whether all organs have stem cells to maintain tissue turnover, many viable stem cells are available. A wide range of adult stem cells has been found in the recent past, including the cartilage, skin, intestine, blood, mammary epithelial cells, and dental pulp [8].

## 2. The Role of Epigenetic in Regenerative Medicine

Genetic and epigenetic mechanisms contribute to changes in gene expression programs without affecting DNA sequence. As a mammal develops, the zygote undergoes numerous differentiation events to produce various types of cells. Epigenetic mechanisms are required to acquire cell-type-specific gene expression programs during differentiation [9]. Qualitatively, epigenetic mechanisms may induce plastic, short-term changes in gene activity and quantitatively, resulting in more stable, long-term changes in gene activity. Multiple cell divisions can be transmitted epigenetic memories in cells that have been stably reprogrammed. Gene regulatory regions can be altered by the presence and activity of ectopically expressed transcription factors. Certain chromatin features have been shown to hinder the process of reprogramming the cell, and therefore, overcoming this barrier is an essential feature of the reprogramming step [10]. Over the past few years, advances in genome-wide association studies have greatly benefited regenerative medicine. Researchers were able to identify multiple somatic mutations affecting epigenome organization and the role of epigenome modifications in the development of cancers. In silico genomic feature annotations and association analysis of genetic association, linkage disequilibrium, and enriched genomic features, referred to as a Bayesian approach, identified more than 200 breast cancer-related signals [11]. Transient, genome-wide epigenomic remodeling has recently been shown to have regenerative capabilities in the formation of organoids and the regeneration of the liver following damage. The study of epigenetic genome modifications involves more than simply the regeneration of tissue and stem cells but also the prognosis and metastasis of multiple types of cancer, specifically relating to tumor microenvironments, immune control, tissue-level mechanical forces, and other cell-intrinsic mechanisms, including transcriptomics and metabolomics [12]. Moreover, epigenetic reprogramming processes essential for developing an embryo from a fertilized egg and establishing cellular totipotency provide clues about epigenetic mechanisms with potential for regenerative therapies [13]. Nevertheless, detailed epigenomic profiling of the human body enabled researchers to determine epigenetic effects on disease development, such as regions of DNA free of nucleosomes specifically targeted by regulatory factors [14]. Also, epigenetic changes have demonstrated a prominent role in the metastasis of various types of cancer, and epigenetic modifications have contributed to the prognosis and diagnosis of various diseases such as neurodegenerative disorders. Therefore, mitochondrial RNAs have other changes apart from the epigenetic changes associated with mitosis [15].

## 3. The Current Used Dental Stem Cells (DSCs) in Epigenetic Modifications

In the connective tissues after birth, there are several multipotent MSCs. These bone marrow multipotent stromal cells were initially shown to proliferate *in vitro* as colony-forming unit-fibroblasts. Some stem cells share similar characteristics among tissues (adipose tissue, liver, and existing blood in the cord). In addition, they have been discovered in the follicle of the newly formed dental embryo and the root apex of growing permanent teeth, in addition to the periodontal ligament and the pulp of permanent and mature deciduous teeth [16]. DSCs have been isolated from various alternative sources of human dental tissues, such as stem cells from apical papilla (SCAPs), human exfoliated deciduous teeth (SHEDs), periodontal ligament stem cells (PDLSCs), and dental follicle precursor cells (DFPCs). Adult stem cells used for regenerative endodontic procedures, such as DFPCs and PDLSCs, can be restricted due to their lack of odontogenic mineralizing properties or, as in the case of SHEDs and SCAPs, their scarcity. A source of SHEDs is the exfoliated pulp tissue of deciduous teeth. Rather than stem cells, pulp tissue is typically used to store the cells obtained from exfoliated or extracted deciduous teeth. SHEDs may be derived incubatively via fluorescence-activated cell sorting and pulp tissue enzymatic digestion and expanded to follow the adequate number. It is relatively easy to obtain these cells, but several investigators have proved harvesting difficult. Some have reported that low volumes of SHEDs could not be obtained because of infection or lack of viable tissue [2]. MSCs are known to have regenerative potential abound in dental tissue. They include porcine DPSCs, SHEDs, PDLSCs, SCAPs, and dental follicle stem cells (DFSCs), which possess easy accessibility. There have been broad properties for DSCs in regenerative medicine due to their potential for osteogenic, adipogenic, and chondrogenic differentiation and their ability to form mineralized tissues. A possible approach will be to use bioscaffolds or biomaterials infused with growth factors. A second tack is to study the effects of natural compounds on dental-derived stem cells, such as polydatin and beer polyphenols. Stem cells in the dental pulp have been established by demonstrating pulp healing postdefect and maintaining homeostasis of related tissues. Porcine DPSC cultures promote endothelial, fibroblastic, and osteogenic (type I collagen, Alkaline phosphatase, osteocalcin, osteopontin, and osteonectin) markers. A variety of bone matrix proteins and odontoblast-specific markers such as dentin sialophosphoprotein are absent from porcine DPSCs tissue, confirming its undifferentiated status. As well as their multilineage differentiation potential, DPSCs can also be identified by the expression of surface antigens. Their surface antigens include STRO-1, CD90, 44, 73, 105, and 271. Although these are not specific markers of DPSCs, they provide further evidence of DPSCs *in vivo*. Characteristics of CD34 and 117 as hematopoietic lineage markers and glia-2, a marker of neurovascular origin, are promoted in DPSCs for the DPSCs' regenerative potential [17]. The current studies of epigenetic regulation of DSCs are summarized in Table 1 in regenerative medicine.

**Figure 1:**
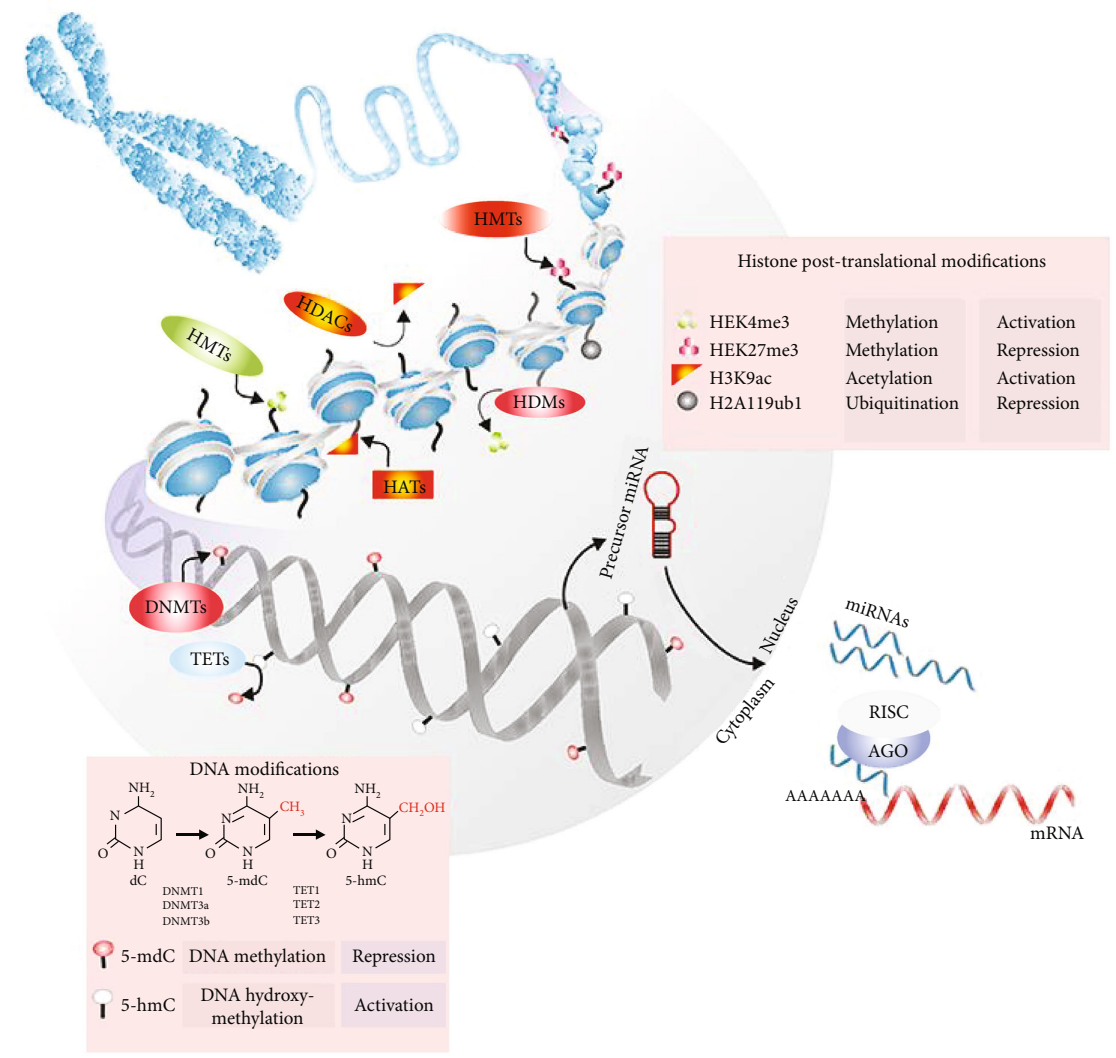
DNA methylation, histone posttranslational modifications (HPTMs), and ncRNAs are the most common epigenetic markers in chromatin remodeling and restructure. A methyl group (-CH3) attach to 5-methyl-deoxy-cytidine (5-mdC) by DNA methyltransferases (DNMTs). The family members of the ten-eleven translocations (TET) can remove DNA methylation marks by converting 5-mdC to 5-hydroxymethylcytosine. Histone methyltransferases and HDACs have been shown to regulate cell methylated, acetylated, and ubiquitinated histone patterns. mRNA can be repressed during transcriptional repression by ncRNAs, such as miRNAs. The Dicer breaks down miRNA precursors in the cytoplasm, and the miRNA is then filled and attached to mRNA targets. Inhibition, degradation, and/or destabilization of the miRNA-mRNA interactions depend on their base pairing [8].

Cell differentiation and development rely on epigenetic modifications that regulate gene expression without altering DNA sequences. Epigenetic influences on embryonic stem cells have gained attention recently [18]. DNA methylation and histone modification as epigenetic modulatory mechanisms have been defined, the most extensively investigated. It has been shown that controlling DPSCs' self-renewal and differentiation can be therapeutic. In the last few years, intensive research has investigated how ncRNAs seem to function as epigenetic regulators of gene expression. Because ncRNAs are implicated in regulating gene expression during health and disease, concluding inflammatory, and reparative processes, they are of particular importance, as a diagnostic biomarker or as part of a dental therapy program; epigenetic modifications may be an effective, promising option [19]. An organism is formed through two remarkably organized procedures: expanding the cell number and changing the phenotypic characteristics with significant spatial-temporal accuracy while growing its organs and tissues. The genetic information for entire cells in an organism is similar. To specify a cell lineage or stimulate cell fate alterations, specific transcription factors must cross-react with each other and apply cross-antagonistic effects. Similarly, stem cell renewal is modulated via epigenetic mechanisms that alter the accessibility of chromatin and determine the direction of cell identity through transcriptional events [8]. Ai et al. showed that DNA methylation patterns are associated with bone formation. They found that DPSCs, DFPCs, and PDLSCs had analogous patterns for DNA methylation. The PDLSCs were more osteogenic-related factors transcriptionally active than DPSCs and DFPCs, exerting a suitable osteogenic capacity both *in vitro* and *in vivo*. Also, DNA methylation modulates the DSCs' odontogenic differentiation by regulating osteogenic differentiation. TET1 inhibited DPSC proliferation and odontogenic differentiation, indicating that demethylation of DNA affects dental tissue regeneration [3]. Epigenetic mechanisms such as methylation of cytosine remain in DNA, changing the posttranslational histone cores, and intercepting transcriptional information and translation can activate epigenetic mechanisms at different levels (Figure 1).

DNA methylation is an epigenetic tag widely studied for its role in transcriptional repression of promoters, chromosome compaction, and cellular memory, among other effects. In particular, DNMTs such as DNMT3a, DNA methyltransferase 3b (DNMT3b), and DNMT1 are involved in these modifications, which modulate the chromatin conformation during embryonic stem cell differentiation and somatic cell reprogramming. Interestingly, stem cells' genomes are largely euchromatic, while the genomes of somatic cells are enriched in heterochromatic conformation [8]. In a study on PDLSCs, Yu et al. revealed that the subpopulation of Alkaline phosphatase ^+^ demonstrated greater CD146 and STRO-1 expression than Alkaline phosphatase ^−^ cells. In addition, some stemness-associated genes were expressed in Alkaline phosphatase ^+^ cells (OCT4, NANOG, and SOX2) compared to Alkaline phosphatase ^−^ cells [20]. To derive PDLSC populations that are homogenous, Alvarez et al. investigated the surface markers, including CD271/140*α*, 51, and STRO-1/CD146. As a result of CD271-positive cells having a more significant dental/osteogenic potential, they were associated with a tremendous increase in osteogenic gene expressions, such as distal-less homeobox 5 (DLX5), bone gamma-carboxyglutamate protein, and runt-related transcription factor 2 (RUNX2) [21]. Researchers found that activated yes-associated protein (YAP) inhibits apoptosis in human PDLSCs, stimulates proliferation, and expedites the cell cycle and retardation of senescence [22]. Another experiment revealed that high glucose levels led to increased DNA methylation levels in PDLSCs, blocking their ability to differentiate into osteoblasts. Nevertheless, 5-Aza-2-deoxycytidine could inhibit the canonical Wnt signaling pathway and increase Alkaline phosphatase, osteocalcin, and osteopontin genes, restoring osteogenic differentiation capacity in PDLSCs [23]. Also, Yu et al. reported that TET1 and 2 reductions cause the hypermethylation of the Dickkopf Wnt signaling pathway inhibitor 1 precursor, which actuated the pathway, upregulation of FasL expression, and betterment the PDLSCs immune regulation properties. TET1/TET2-reduced PDLSCs demonstrated remarkable upregulation of therapeutic ability in the colitis mice model [24].

### 3.1. Postnatal Human Dental Pulp Stem Cells (DPSCs)

DPSCs are isolated from a postnatal human dental pulp to regenerate a reparative dentin-like complex and differentiate into different cell types. These cells are unique among stem cells because they can be separated into diverse cells, including neural progenitors, odontoblasts, melanocytes, chondrocytes, osteoblasts, smooth muscle cells, and adipocytes. DPSCs are an auspicious tissue type for dental tissue engineering and craniomaxillofacial regeneration due to their high proportion of prevalence, low morbidity, well differentiation, and biomaterial toleration. In addition to osteogenic and adipogenic differentiation, DPSCs also show neurogenic differentiation, similar to SHEDs, making them very attractive for clinical use [25]. Since the DPSC high differentiation plasticity marks them an ultimate stem cell source for cellular treatment, regeneration, and engineering of tissues for multiple disorders, they are currently being explored. Stem cells originate from the dental pulp of permanent teeth, which contains wisdom teeth surgically removed that do not contribute to the occlusion of permanent teeth. Genetic and congenital disorders are often characterized by defects in the fetal or postnatal stages [16].

DPSCs usually differentiate into various cell types and have also proven to retain many of their characteristics after cryopreservation for two years. As a result, many studies are being conducted on the DPSC differentiation and their clinical potential. The multidirectional differentiation capacity and the easy accessibility of DPSCs make them an excellent candidate for use in tissue engineering and disease. Studies have demonstrated DPSC formation of an immunocompromised complex resembling dentin and pulp. DPSCs in scaffold-free and prevascularized microtissue spheroids may also effectively regenerate vascularization in dental pulp tissues. Also, they could provide a model for dentin regeneration and the treatment of endodontics. The potential for clinical application of DPSCs goes beyond treating dental problems to treating other medical disorders, such as craniofacial defects, nervous system injuries, muscle regeneration, osteoarthritis, myocardial infarction, Alzheimer's disease, diabetes, Parkinson's disease, liver diseases, and stress urinary incontinence [1]. Periodontal disorders, hypodontia, enamel development, and odontogenic differentiation have been associated with epigenetic changes. Duncan et al. presented research on the potential therapeutic potential of inhibiting histone deacetylases and DNA methyltransferases in dental pulp as a regenerative endodontic application. It has been shown that permissive chromatin associated with transcriptional upregulation is instrumental in developing DPSCs into mature odontoblasts. However, there is still limited knowledge about epigenetics and how it leads to specialized cell lineages in DPSCs, despite some progress [25]. Schwann cell markers are expressed after incubation through c-Kit^+^/STRO-1^+^/CD34^+^ DPSC induction, which was demonstrated by Carravale et al. The incorporation of the DPSC collagen scaffold resulted in sensory neurons being regenerated and myelination occurring in the rats' sciatic nerve injury model [26]. Alraies et al. recognized alterations between high (A3)/low (A1 and 2) proliferative ability DPSC populations [27]. A paper by Young et al. elucidated that murine DPSC clones could differentiate into oligodendrocytes and neuron-like cells *in vitro*. Interestingly, only those DPSCs that express remarkable levels of nestin gene expression differentiate successfully into neurofilament-positive neuron-like cells and microtubule-associated protein 2 [28]. As a result of transplantation of CD146^+^ cells into immune-compromised beige mice, Matsui et al. found that dentin/pulp-like structures formed. Moreover, CD146^+^ cells also possessed higher mineralization properties compared to nonseparated cells, CD146^−^ or 146^+/-^ cells. DPSCs transplanted with human mitochondria are immunohistochemically detected to contain dentin matrix protein-1 (DMP1), dentin sialophosphoprotein, and dentin matrix protein-2 (DMP2) [29]. The induction of c-Kit^+^/STRO-1^+^/CD34^+^ DPSCs exhibited superior levels of commitment than that of DPSCs c-Kit^+^/STRO-1^+^/CD34^−^, which may be evidenced via *β*-III tubulin expression and the shift from neuron-like shapes and appearance to spheroid-like appearances [30].

In long-term culture, production of SA-*β*-gal and biomarkers concluding p16, 21, interleukin- (IL-) 1*β*, 6, 8, and growth-related oncogene alpha (Gro*α*) was shown to increase for mobilized DPSCs with age as determined by their revulsive reaction to the colony-promoting factor of granulocytes from different donors. A model of ischemic hindlimb damage and ectopic teeth roots revealed that aged mobilized DPSCs had the similar regenerative potential to young mobilized DPSCs [31]. Salkin et al. demonstrated that transforming growth factor-beta 1 (TGF-*β*1) transfection promotes proliferation and prevents apoptosis and cellular senescence, suggesting a potential therapeutic intervention. They proposed that the overexpression of TGF-*β*1 along with gene transmission might lead to the enhanced DPSCs' biological abilities and replace the external delivery of recombinant proteins into the cells [32]. 5-Aza was used by Nakatsuka et al. to assess the Myod potential of DPSCs in mice. DNA demethylation caused via 5-Aza and forced Myod-1 expression stimulated expression of transcription factors related to muscle-specific [33]. The results of Paino et al.'s study showed that histone deacetylase 2 (HDAC2) silencing could increase the expression of osteocalcin and bone sialoprotein in DPSCs, similar to the effect of valproic acid [18]. The expression profile of circular RNAs was revealed in DPSCs during odontogenic differentiation by Chen et al. 43 circular RNAs were upregulated during dental differentiation, while 144 circular RNAs were downregulated. Signaling pathways regulating pluripotency in MSCs are abundant in these differentially expressed genes, such as the TGF and Wnt signaling pathways [34].

Somatic cells' epigenome is improved with heterochromatin, permanently silencing more genes. Stem cells have a particular genome in euchromatic conformation; mainly, differentiated cells' genome is a mixture of euchromatic and heterochromatic forms. In addition, DNA methylation levels in DPSCs are low both *in vitro* and *in vivo*, and histone acetylation levels are high in DPSCs, which may weaken the interaction between chromatin and DNA, allowing the expression of genes to proceed. Conversely, loss of acetylation mediated by HDAC results in a closed heterochromatin conformation, suppressing transcription. Recent findings have associated histone methylation with chromatin remodeling in DPSCs. Trimethylation marks of H3 are the most characterized, and these marks serve as gene activators for transcriptional activation and repressors for transcriptional silencing for transcriptional repression. Moreover, cell stemness and differentiation genes contain bivalent histone methylation marks, which can activate H3K4 trimethylation and repress H3K27 trimethylation histone methylation in DPSCs. The bivalent domain-containing genes in the stem cells would allow them to respond more rapidly to environmental changes by repressing specific genes at the same time while activating others [35]. DNA methylation, histone modifications, and ncRNAs are crucial in controlling DPSCs' fate. Genetic control is mediated by signaling pathways and transcription factors, and epigenetic control is mediated by DNA methylation, histone modifications, and ncRNAs. The manipulation of DPSCs' fate toward pulp–dentin regeneration is possible with an epigenetic modulation understanding in DPSCs [17]. Among the most well-researched epigenetic modifications, DNA methylation is often associated with gene silencing and stem cell fate regulation. Several studies have also found particular regulatory effects of DNA methylation in DPSCs [1]. Recent studies have observed complex epigenetic networks for porcine DPSCs, including long noncoding RNAs (lncRNAs), microRNAs (miRNAs), and DNA methylation. The lncRNA G043225 stimulates odontogenic differentiation through direct interactions with fibrillin 1 and miR-588. lncRNA H19 generally suppresses DNMT3b activity, decreases the DLX3 methylation level, and therefore causes the advancement of porcine DPSC odontogenic differentiation. In the same way, miR-675 inhibits the DNMT3b-mediated methylation of DLX3 in DPSCs for promoting human DPSC odontogenic differentiation. In addition, colon cancer-associated transcript 1/lncRNA increases cell differentiation and proliferation by suppressing the miR-218 signaling pathway [17]. H3K4 trimethylation activates remodeling acetylases of histone and enzymes along with the increment of transcription. In contrast, histone H3K27 trimethylation functions in the opposite way. In this regard, H3K27 trimethylation and H3K4 trimethylation conduct on progressive genes to provide bivalent domains genome together. It is believed that the differentiation-relevant genes that up- and downregulate through opposing modifications of the associated histones are “locked away” but kept “poised,” ready for activation once the appropriate signals come. Almost half of the bivalent domains in the mouse genome have been associated with connecting areas for the OCT4, NANOG, and/or SOX2 transcription factors. Conspicuously, most domains ultimately revert to H3K27 trimethylation or H3K4 trimethylation based on their lineage [12].

### 3.2. Dental Pulp-Derived Mesenchymal Stem Cells

Teeth are composed of several types of cells, including odontogenic and undifferentiated progenitor cells, within a remarkably vascularized connective tissue center and undifferentiated stem cells, which also include multipotent osteoblasts that have both significantly proliferative properties *in vitro* as well as *in vivo*. During early embryonic development, mesenchymal crest cells transfer to the branchial arches of the nervous system, indicating that the MSCs that make up dental pulp are descended from neural crest cells. The three prominent human teeth are deciduous, permanent, and supernumerary, contributing to developmental pathways and morphological properties; however, they vary molecularly. In a study of dental pulp cells isolated from these teeth, multipotent MSCs were observed. However, several differences were observed at the molecular and cellular levels. Rodent nerve injury models showed that these transplanted cells inhibited apoptosis and inflammation that interfered with repairment while differentiated into oligodendrocytes, which are mature, to stimulate neuroregeneration [16]. Reduction expression of STRO-1 and regulation of transcription factors, NANOG, OCT4, and nestin, was observed with an upregulation in gingival-derived mesenchymal stem cell (GMSC) passage by Ranga Rao and Subbarayan [36].

### 3.3. Human-Exfoliated Deciduous Teeth Stem Cells (SHEDs)

Deciding teeth exfoliate spontaneously upon extrusion by their permanent successors or are surgically removed before breaking out permanent successors. These extracted or exfoliated deciduous teeth involve residual pulp tissues concluding dental MSCs, first discovered as SHEDs approximately 2 decades ago. They may be accessed with a slightly invasive process. The SHED multipotency is indicated by their shared adipose, osteogenic, and chondrogenic origins with BMSCs. SHEDs express MSCs and embryonic stem cell biomarkers, lack hematopoietic signaling biomarkers, and involve CD11b/c and 45. Patient-derived SHEDs are ideally constructed from a child in the process of a genetic condition that does not affect the child's natural teeth. When an individual has twenty deciduous teeth, the maximum number of teeth that could hypothetically be attained from a described child in the process of genetic disorder is twenty. As a result, there are twenty chances for SHEDs to be collected from a child. This is deciduous teeth benefit, which is more common in children, over wisdom teeth which differ in that adults have a maximum of four sets. Consequently, patients with genetic disorders may have fewer deciduous teeth to establish disease models derived from SHEDs. This represents a severe disadvantage. For these reasons, professional dental care for children with severe consequences is vital for their quality of life, oral health, and setting up patient-derived SHED models of genetic conditions [16]. According to Inada et al., two of the five primarily isolated SHEDs had higher OCT3/4 expression and had higher Alkaline phosphatase activities. These two lines proliferated faster and were simpler to program into induced pluripotent stem cells (iPS cells) [37].

## 4. The Role of Histone Modifications in DSC Differentiation

**Figure 2:**
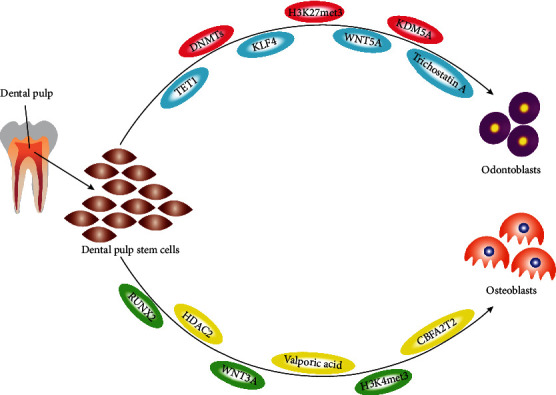
The pathways of dental pulp stem cell (DPSC) differentiation. Yellow and red ovals block differentiation pathways, and green and blue ovals stimulate the differentiation of DPSCs. Abbreviations: DNMTs: DNA methyltransferases; TET: ten-eleven translocations; H3K27met3: histone H3 lysine 27 trimethylations; H3K4met: histone H3 lysine 4 trimethylation; KDM5A: lysine demethylase 5A; HDAC2: histone deacetylase 2; KLF4: Krüppel-like factor 4; WNT5A: Wnt family member 5A; WNT3A Wnt family member 3A; RUNX2: runt-related transcription factor 2.

Heterochromatin modifications, including histone modifications, are also essential factors in the fate of DSCs. Lysine demethylase 3B (KDM3B) increases osteogenic differentiation of SCAPs. Lysine demethylase 4B (KDM4B) removes H3K9 trimethylation via attaching to DLX stimulators, contributing to aim gene expression [3]. It was reported by Yang et al. that DLX5 and KDM4B are modulated by SCAP-positive feedback loop. Moreover, DLX5 increases the osteogenic differentiation genes DMP1, dentin sialophosphoprotein, and osteopontin, encouraging their expression. The nude mice model investigation also implied that DLX5 promotes osteogenesis by upregulating KDM4B in SCAPs. At the same time, lysine demethylase 5A (KDM5A) inhibits the DPSC capacity to differentiate to the dentin morphotype via the elimination of H3K4 trimethylation from the dentin sialophosphoprotein, DMP1, and stimulators of osteocalcin [38]. Transcription factors can modulate access to target genes by altering at least 12 amino acid residues in histones. Also, acetylation and methylation, two histone modifications, are vital for various biological procedures, concluding determination of cell fate and transcriptional modulation [8]. It is also possible to control gene expression epigenetically through ncRNAs, including lncRNA, siRNA, and miRNA; however, ncRNA amount exceeds mRNA transcription coding. Small interfering RNA (siRNA)/miRNAs function by controlling them for aiming mRNA strands. The marked mRNA strands are then cleaved. It is not clear with certainty where miRNAs function; nonetheless, a minimum of one-third of genomes are expected to be modulated via the coding of miRNAs. Both miRNAs and siRNAs process several targets, but the former has just one aim. The extent to which miRNAs affect gene expression is still being determined [19]. In addition, several miRNAs may be involved in regulating differentiation in these dental tissues, including miR-99a, miR-210, and miR-218. Considering the relation between miR-218 lower levels and higher RUNX2 levels, it would appear that the expression of miR-218 is essential to managing RUNX2 expression, a transcription factor crucial for osteogenic differentiation. Findings suggest that miR-218 modulates RUNX2 expression to regulate osteogenic sequences in human dental tissue-derived MSCs. The regulation of osteogenic differentiation capacity by miRNA differential profiles remains to be determined. The expressions of miR-101 and -21 are implicated in osteogenic differentiation of periodontal ligament stem cells; both miRNAs enhance the mineralization ability of periodontal ligament stem cells by regulating the expression of periodontal ligament associated protein 1 (Figure 2). Meanwhile, several miRNAs were distinctively expressed among the odontoblast differentiation of DPCs, such as miR-32, -586, and -885-5 (Figure 2). Further, increased expression of noninductive stemness markers, such as osteopontin, dentin sialophosphoprotein, osteocalcin, and dentin sialophosphoprotein, is maintained by overexpression of p300. These results indicate that p300 interacts with stemness markers and conducts a vital role in noninductive conditions. It has been shown that acetylation of H3K9 increases in the genes' specific regions associated with odontogenic potential when p300 is overexpressed (Figure 2) [8]. Nakatsuka et al. revealed that miR-34a suppressed the process of osteogenesis via inhibiting the cell cycle and proliferation of cells [33] and that the miR-34a suppression could expedite this process BMSC osteogenesis. In contrast, Xin et al. implied that the upregulation of miR-34a might facilitate the process of BMSC osteogenesis and regression of proinflammatory cytokine by aiming tumor necrosis factor-alpha (TNF-*α*) [39].

## 5. The Role of Dental Stem Cell's Self-Renewal and Differentiation in Regenerative Medicine

DSCs possess the potential for self-renewal and multidifferentiation similar to MSCs. Research and clinical advances have been made in dental pulp regeneration during the past few years. In regenerative endodontics, several strategies have been proposed, with different scaffolds, growth factors, and stem cells, emphasizing important aspects of dentistry such as disinfection and dentin conditioning [40]. DPSCs were first reported in 2000. Since then, they have become the most commonly used DSCs for developing cell-based therapies for dental and systemic diseases. Dental pulp tissue, which can be harvested from extracted teeth, appears to contain potential stem cells that can be used in clinical applications in the future. A laboratory led by Yaegaki has recently induced a new type of cell, hepatocytes, from dental pulp cells isolated from full-grown wisdom teeth and exfoliated deciduous teeth [41]. The researchers from Nakashima et al. recently published a pilot study that demonstrated the potential benefit of mobilizing DPSCs for pulp regeneration in human teeth that have undergone pulpectomy. Clinical and laboratory evaluations revealed that the pulp responded positively with no toxicity despite the short sample size. An ongoing randomized, controlled study in a single center is currently being conducted to evaluate the efficacy of using autologous SHEDs to revitalize young necrotic permanent teeth [42]. The clinical applications of DPSCs have been demonstrated in several studies, showing that the cells can promote bone regeneration and neo-bone formation in cranial defects, suggesting their potential impact on regenerative medicine, including bone conditions [43]. Additionally, other studies have examined how DSCs differentiate into nerve tissues and pancreatic cells and their efficacy as a source of iPS cell proliferation [44, 45]. DSCs have recently become more popular due to the extension of dental applications to other fields of medicine. Researchers continue to explore the potential of DSCs to differentiate other cell types. This may provide better quality treatments for diseases such as diabetes mellitus, Alzheimer's, Parkinson's, myocarditis, and other devastating conditions [46].

## 6. The Role of Pharmacological Agents in Epigenetic Modifications

Molecular design of multitargeting epigenetic agents has become a popular method for developing epigenetic therapies by specifically targeting several unrelated cellular targets at once (at least one of which is the epigenetic enzyme). Multiaction drugs simplify treatment regimens by reducing adverse drug reactions, reducing the likelihood of drug resistance, and facilitating easy administration. Zinc-dependent HDAC inhibitors are commonly used to target more than one cancer-related target. Additionally, methyltransferase and demethylase enzymes are also commonly targeted, as are acetyllysine-binding bromodomains [47]. There are pharmaceutical agents which can generally change histone acetylation epigenetically. These molecules, known as histone deacetylase inhibitors (HDACis), have changed gene transcription, induced pleiotropic cell effects, and affected stem cells fate. Several types of HDACi effectively induce differentiation, proliferation, and anti-inflammatory properties, concluding with valproic acid, trichostatin A, and butyric acid. Recent studies have demonstrated that HDACis perform an epigenetically vital function in regulating DPSC differentiation and self-renewal by working in a balance with histone acetyltransferases. This HDACi suppresses N-terminal deacetylation located in the histone tail within the nucleosome, leading to modification of chromatin structure and increased transcription. There are eighteen human HDAC enzymes, each possessing a distinct, complex, but often overlapping role, which has yet to be fully explained. HDACs express themselves in the cytoplasm, nuclear (noncytoplasmic), or tissue-restricted (cytoplasmic) locations. Consequently, pan-HDAC inhibitors that target all 18 enzymes are being explored more than isoform-specific suppressors. Some pan-HDACis were performed to accelerate differentiation and dental pulp cell population mineralization and dedifferentiate immature stem cell populations in specific environments and concentrations [2]. The histone posttranslational modifications, which occur on particular amino acid histone proteins remains, are another critical epigenetic mechanism. The modification types in histone tails contain methylation, deimination, acetylation, sumoylation, parylation, ubiquitination, isomerization, citrullination, and phosphorylation. The modifications impact transcription, replication, DNA repair, and chromatin structure. These modifications may also serve as potential targets for anticancer drugs [47]. Several clinical trials combining HDAC inhibition with protein kinase inhibition have led to the concept of combining both actions into one compound. Interestingly, Zang et al. have shown that HDACi combined with pazopanib results in positive antitumor effects [48]. Based on the osimertinib structure, another epidermal growth factor receptor (EGFR) inhibitor that is approved, researchers designed and synthesized dual inhibitors of HDAC and EGFRs [49]. In comparison with vorinostat (approved HDAC inhibitor), some of the designed compounds inhibited HDAC at a greater level. These were regarded as moderate to low EGFR inhibitors. Another approach was to pair ruxolitinib with vorinostat to create dual Janus kinase (JAK)-HDAC inhibitors. As a result of a pyrazole substituted pyrrolopyrimidine (compound number 24) being highly potent and selective against a panel of 97 kinases, Yao et al. reported IC50 below 20 nM for inhibiting JAK1 and HDAC1 and 2, 3, 6, and 10 [50]. In an experiment by Kuang et al., hypoxia-primed porcine DSCs were implanted with a synthetic polymer to construct a three-dimensional rat model in an in situ model. Histologically pulp-like tissues were generated and vascularized. Another group of scientists conducted BMP2-treated DPSC culture onto the amputated dog canine pulp, which are autogenously transplanted [51]. It was supposed that preconditioned porcine DPSCs would guide differentiation precisely and ensure optimal functional regeneration of pulp. Autologous SHEDs must undergo long-term follow-up despite the promising and cheerful results obtained by Xuan et al. Additionally, allogenic DPSCs are more effective and safer than self-derived ones. Further research is needed on allogenic DPSCs [52].

## 7. Conclusion

It is crucial to summarize the current epigenetic cues to advance clinical research into DSCs. Epigenetic modifications influence several critical signal pathways, ultimately responsible for DSC fate. In addition, by deciphering the epigenetic code of DSCs, regenerative therapies could be directed at DSCs from bench to bedside, thus making DSCs more accessible. Bioengineering relies heavily on stem cells, so it is essential to elucidate how cell fate regulation affects differentiation. Enhancer Of Zeste 2 Polycomb Repressive Complex 2 Subunit (EZH2) blocks the differentiation of bone, muscle, neural, and hematopoietic precursor cells in MSCs. As well as inhibiting NANOG activity in embryonic stem cells, EZH2 suppresses the activity of POU class 5 homeobox 1 - two pluripotency genes - proposing that modifiers of histone are phase- and cell-specific and act differently based on the cell type and differentiation state. To reach clinical application, several regulatory obstacles must be overcome. Firstly, it is vital that off-target effects are considered. Almost all HDACis, like TSA and valproic acid, are paninhibitors with no specific selectivity, so they upregulate target proteins. In immunomodulation, for instance, the expression of anti-inflammatory factors can be overexpressed while an individual miRNA upregulates proinflammatory cytokines. Secondly, epigenetic therapeutics must be thoroughly investigated and screened to minimize unwanted effects before being used in clinical settings to minimize the possibility of neoplastic transformation during regeneration. Lastly, technical and financial support must be endorsed to bank DPSCs and reserve the “biological insurance.” Also, it is required to develop standardization and optimization of manufacturing protocols to guarantee the cell source quality at all stages of cryopreservation, isolation, collection, and expansion.

## 8. Future Direction

Stem cells are essential for dentin-pulp regeneration, signaling molecules that regulate cell fate, scaffolds that provide a favorable microenvironment, and stem cells with pluripotency capacity. DPSCs are regulated by complex epigenetic networks of histone modifications, ncRNAs, and DNA methylation that promote migration, self-renewal, and multidifferentiation. Epigenetic regulation in these processes will help improve DPSC migration, self-renewal, and multidifferentiation during the regeneration of pulp tissues. There have been positive results with HDACis on bone regeneration in animal models, including trichostatin A. Therefore, HDACis could regenerate pulp and dentine *in vivo*. Today, DNMT inhibitors and ncRNAs do not exhibit any regenerative potential *in vivo*. A suitable prospect is to understand the promising functions of epigenetic modulation in DSC fate and identify novel therapeutic targets for DSC-mediated regeneration. It is critical to assess how to ensure the DSC stemness in standard culture conditions. During long-term cell culture, DSCs may lose some of their potential. Diomede et al. reported that 5-Aza induced the GMSC differentiation into embryonic lineages in 48 hours. The GMSCs exhibited three germ layers and secreted markers related to embryonic development after being treated with 5-Aza. According to the above results, future translational medicine can benefit from epigenetic regulation. Additionally, it has been shown that 5-Aza treatment induces the differentiation of GMSC into different embryonic lineages other than neural precursor cells post extended expansion, indicating that 5-Aza may have a potential role in the preservation of DSCs stemness in the future. DNMT suppression increased Krüppel-like factor 4 (KLF4) levels and accordingly enhanced the rate of DSCs odontoblastic differentiation. Furthermore, stem cell differentiation can realize this great potential for regenerative medicine in specific tissues in the future. While there is a significant amount of research in domains such as delivery methods, off-targets, and neoplastic transformation, these issues must be addressed before epigenetic strategies are optimized for dentin-pulp regeneration. Small molecules can be used to manipulate epigenetic factors of DPSCs to promote differentiation and regeneration in the search for functional pulp regeneration approaches.

## Figures and Tables

**Table 1:** The current studies of epigenetic regulation of dental stem cells in regenerative medicine.

Cell type	Method	Outcomes	Ref/year
Induced pluripotent stem cell (iPS cell) generation from dental pulp stem cells (DPSCs)	A reprogramming scheme was investigated for iPS generation from DPSCs	Sufficient iPS cell generation from DPSCs, improving clinical and industrial utilization of iPS knowledge to use in therapies	[53]/2018
DPSCs	Examination of regulating Notch/Wnt signaling and stimulation of adipo-/osteocytes differentiation in DPSC cell lines	Wnt signaling could participate in the safer progression and lower destructive reprogramming platforms in DPSCs to utilize in cell therapy	[35]/2020
DPSCs and bone marrow-derived mesenchymal stem cells (BMSCs)	Examination of similar genetic and epigenetic mechanisms between the osteogenic differentiation of DPSCs and BMSCs	Common epigenetic and genetic mechanisms are concluded in the osteogenic differentiation of BMSCs and DPSCs	[54]/2021
Human MSCs	Identification of miR-34a aims protein systems as an osteoblastic regulator of [51] human MSC differentiation	miRNA-34a showed particular dual modulatory impacts on both proliferation and differentiation of human MSC. Additionally, miR-34a suppression could be a novel therapeutic approach for increasing the formation of bone tissue	[55]/2014
BMSCs were isolated from C57/BL mice	Investigation of miRNA function in the regulation of osteogenesis procedure in the inflammatory condition	miR-34a reverses proinflammatory cytokine effects and stimulates osteogenic differentiation, exhibiting that therapy based on miR-34a might be a suitable method for stimulating the regeneration of bone tissues	[39]/2019
Human DPSCs	Evaluation of different differentiation circumstances. K^+^ channels initiation is assumed to modulate the Ca^2+^ content, which is intracellular, tolerating to change cell cycle to human DPSC differentiation induction	Epigenetic reprogramming and cell cycle regulation via a promotion with remarkable K^+^ facilitated differentiation of human DPSCs into neuron-like cells. Hence, human DPSCs have the practical function as neuron-like cells via alteration of cells cycle	[56]/2020
Human DSCs	Investigation regulating the specification of signals tissue and lineage of cells epigenetically through evaluation of miRNA activity behind dental stem cells (DSCs)	miRNA-modulated pathway for the human DSC differentiation and a chosen network of miRNAs that control DSC osteogenic differentiation	[57]/2014
DPSCs	Investigation of immunomodulatory abilities of DPSCs by cocultured from elderly and young donors	Decrement of IL-6 and HGF expressions are necessary for the bone and dental tissue regeneration and downregulate highly in elder DPSCs	[58]/2021
DPSCs	Assessment of multipotential differentiation abilities of DPSCs	Ferutinin activated and promoted osteogenic differentiation of DPSCs, as a promising effective stem cell therapy for osteoporosis	[59]/2020
DPSCs	Explore the osteogenic, adipogenic, and resistance to oncogenic transformation of DPSCs in comparison with BMSCs	Several epigenetic factors widely implied tumorigenesis lineage and commitment, which might be considered when progressing stem cell therapeutic uses	[4]/2019
Periodontal ligament stem cells (PDLSCs)	To characterize DPSCs and their mechanism of differentiation cells from human DPSCs and PDLSCs, explore a miRNA array based on LNA	miR-720 reduced DPSC proliferation as distinguished via immunocytochemical assessment against ki-67 and stimulated odontogenic differentiation, like Alkaline phosphatase and mRNA levels of osteopontin. Also, outcomes demonstrated that miR-720 is a modulator miRNA for the DPSC differentiation	[60]/2013
Human DPSCs	Odontoblast-related genes changes were explored epigenetically via the alteration of the mitogen-activated protein kinase (MAPK) signaling pathway in cell lines	Cell proliferation downregulated response to MS-275 using, while it did not affect cytotoxicity in 5 and 10 nM and induced odontoblast-like cells differentiation	[61]/2020
DSCs	Evaluate KDM6B knockdown in DSCs and its effect on Alkaline phosphatase function and mineralized nodules formation	Outcomes exhibited participation of HDMs in the epigenetic modulation of odontogenic differentiation of DSCs. Lysine demethylase 6B (KDM6B) may indicate a promising beneficial aim in the tooth repairment and regeneration of craniofacial tissues	[62]/2013
DPSCs	N^6^-methyladenosine (m^6^A) methylation biological actions were assessed in DPSCs	m^6^A methylated hallmarks in DPSCs and modulatory participation in the cells cycle. It can use as a therapeutic approach in vital pulp therapy	[63]/2021
Mesenchymal stem cells (MSCs) derived from bone marrow and dental tissues	Address the function of long noncoding RNAs (lncRNAs) in osteogenesis modulation of MSCs derived from bone marrow and dental tissues. Also, lncRNAs as therapeutic aims for MSC-related diseases were investigated	lncRNAs involved in the bone marrow and dental tissue-derived MSCs' osteogenic differentiation could aim as prognosis and therapeutic parameters. Nevertheless, the lncRNA precise actions remain elusive	[64]/2018
MSCs	Investigate trichostatin A effects on osteogenic differentiation and resolve inflammation on MSCs derived from inflamed and normal gingival tissues	Anti-inflammatory properties and stimulation repairment of periodontal tissue were shown via trichostatin A as a candidate for therapeutic approaches in repairing periodontal tissues	[65]/2020
DPSCs	Explore genome-wide gene expression microarray and DNA methylome investigation to clarify molecular changes by DNA methylation alterations correlated with DPSC exposure to ethanol	Findings proved that significant alcohol usage might affect cellular processes that cause decreased mineral deposition, resulting in osteoporosis/-penia, dental abnormalities, and hallmark conditions for different fetal conditions induced via alcohol	[23]/2016
DPSCs	Examine the interference with N6-adenosine-methyltransferase 70 kDa subunit (METTL3) in DPSCs inhibits cell proliferation and osteogenic differentiation	Findings provide new ideas for using stem cells in clinical applications and for treating metabolic bone diseases by altering epigenetic modifications	[66]/2021
Human DPSCs	H19 mechanisms and impacts were assessed in human DPSC odontogenic differentiation	Provide novel visions of how the S-adenosylhomocysteine hydrolase (SAHH)/H19 axis functions in the odontogenic differentiation of human DPSCs. It would help develop therapies for the regeneration of dentin following stem cells	[67]/2018
Human dental follicle stem cells (DFSCs)	Examine Enhancer Of Zeste 2 Polycomb Repressive Complex 2 Subunit (EZH2) and histone H3 lysine 27 (H3K27) trimethylation expression during osteogenesis of human DFSCs	EZH2 promoted the Wnt/*β*-catenin pathway by modulating the level of H3K27 trimethylation on stimulators of genes in these signaling pathways	[68]/2018
Human DPSCs	Assessment of trichostatin A on differentiation and proliferation of odontoblast along with its capacity in the forming of dentin and odontoblast differentiation in vivo during tooth progression	Trichostatin A conducted vital function in odontoblast differentiation and proliferation of human DPSCs in dental progression phases	[69]/2013
DSCs	Investigate the DSC niche cell types and the miR-200 class effects on the fates of DSCs	miR-200 modulates signaling pathways necessities for cell differentiation, progression of the cell cycle, and DSC niche maintenance	[70]/2021
DPSCs	Evaluate the regulatory role of Krüppel-like factor 2 (KLF2) during osteoblast DPSCs differentiation via assessing the KLF2 levels and autophagy-related molecules in cells	Chromatin immunoprecipitation evaluation showed that the functional epigenetic biomarkers and KLF2 were elevated in the stimulator region of autophagy-related 7 (ATG7)	[71]/2020
Human DFSCs	Investigate genes' functions and regulatory mechanisms (HOXA transcript antisense RNA, myeloid-specific 1 (HOTAIRM1), and homeobox A (HOXA)) in human DFSCs	HOTAIRM1 stimulated the human DFSC osteogenesis via modulating homeobox A2 (HOXA2) through DNA methyltransferase 1 (DNMT1). HOXA2 exhibited necessary actions in human DFSCs, same as HOTARIM1. Nonetheless, the HOTARIM1 modulatory pattern within the HOXA group remains unknown	[72]/2020
Mice DPCs	Identify Spalt-like transcription factor 1 (SALL1), polarizing and secretory odontoblasts, in vivo	SALL1 effectively modulates the odontoblast lineages commitment via connection with runt-related transcription factor 2 (RUNX2) and straight activation of transforming growth factor beta-2 (TGF*β*-2) at an initial phase	[73]/2021
Human DPSCs and BMSCs	Investigate corepressor CBFA2/RUNX1 partner transcriptional corepressor 2 (CBFA2T2) expression was remarkably increased in response to BMP2 treatment during osteogenic differentiation of human DPSCs and BMSCs	CBFA2T2 is required for BMP2-induced osteogenic differentiation of MSCs by inhibiting euchromatic histone lysine methyltransferase 1- (EHMT1-) mediated histone methylation at RUNX2 stimulator	[74]/2018
Human DPSCs	Histone H3 lysine 4 (H3K4) trimethylation and H3K27 trimethylation spatiotemporal patterns were examined in the mice model. Human DPSCs induced during odontogenic differentiation, H3K27 trimethylation demethylases (UTX and JMJD3), and H3K4 trimethylation methylases	During dental MSCs differentiation, Wnt family member 5A (WNT5A) transcription activities were modulated via stability among H3K27 trimethylation/H3K4 trimethylation, Jumonji domain-containing protein-3, and H3K4 trimethylation methylase stimulator	[75]/2018
Human DFSCs	Explore the osteogenic differentiation of human DFSCs and chromodomain helicase DNA binding protein 7 (CHD7) expression	CHD7 regulates the osteogenic differentiation of human hepatocytes by modulating the transcription of parathyroid hormone 1 receptor (PTH1R). Also, overexpression of PTH1R partially restores osteogenic differentiation in CHD7-knockdown human DFSCs	[76]/2020
Human DPSCs	Investigate the photobiomodulation therapy (PBMT) role on viability, human DPSCs migration, and its correlation to epigenetic mechanisms such as acetylation of histones	There is a correlation between PBMT and high viability and human DPSCs migration, related to the histone acetylation upregulation. Also, PBMT is an appealing adjuvant therapy for regenerative endodontic treatment	[77]/2020
Human DPSCs	Evaluate epigenetic reprogramming via the histone deacetylase 3 (HDAC3) and histone deacetylase 2 (HDAC2) selective inhibitors, MI192, to stimulate the osteogenic capacity of human DPSCs for bone regeneration	By reprogramming epigenetic factors of hDPSCs with HDAC2- and HDAC3-specific inhibitors, MI192 improves osteogenic differentiation, implying the feasibility of this method for bone augmentation	[78]/2021
Dental pulp and dental follicles	Differentiation profiles and epigenetic states of dental follicle/dental pulp, two odontogenic neural crest-derived ancestor populations, were examined	The results showed to highlight the crucial function that epigenetic regulation conducts in the odontogenic terminal differentiation neural crest cells	[79]/2013
PDLSCs	Examine the periodontal regeneration effect on the heterogeneous nuclear ribonucleoprotein L (HNRNPL) mechanism in the osteogenesis of PDLSCs induced by strontium chloride (SrCl_2_)	There may be some implications for the treatment of periodontitis patients who have osteoporosis simultaneously by understanding the different functions of HNRNPL and SET domain containing 2, histone lysine methyltransferase (SETD2)	[80]/2019
PDLSCs	Assess the lysine demethylase 6A (KDM6A) function in chondrogenic differentiation of PDLSCs and the underlying mechanisms related to epigenetic	In the destruction of inflammatory tissue such as osteoarthritis, it was expected an improvement in MSC-mediated regeneration of cartilage through upregulation of KDM6A or the use of EZH2-inhibitors	[81]/2018
Cranial neural crest cells	Kat2a and 2b genes function as histone acetyltransferases and were examined in the progression of craniofacial in zebrafish and the *Gcn5* in mice	As a result of regulating H3K9 acetylation, these outcomes proposed that Kat2a and 2b are essential to the growth and cartilage and bone differentiation in both mice and zebrafish	[82]/2018

## Data Availability

All data are included in the article.

## Abbreviations

RNA: Ribonucleic acid
ncRNAs: Noncoding RNAs
DSCs: Dental stem cells
DNA: Deoxyribonucleic acid
MSCs: Mesenchymal stem cells
DPSCs: Dental pulp stem cells
SHEDs: Stem cells from human exfoliated deciduous teeth
PDLSCs: Periodontal ligament stem cells
DFPCs: Dental follicle precursor cells
BMSCs: Bone marrow-derived mesenchymal stem cells
SCAPs: Stem cells from apical papilla
GMSCs: Gingival-derived mesenchymal stem cells
CD: Cluster of differentiation
DNMTs: DNA methyltransferases
TET: Ten-eleven translocations
PTEN: Phosphatase and tensin homolog
AKT: Protein kinase B
5-Aza: 5-Azacytidine
H3K9: Histone H3 lysine 9
H3K27: Histone H3 lysine 27
H3K79: Histone H3 lysine 79
H3K4: Histone H3 lysine 4
H3K36: Histone H3 lysine 36
mRNA: Messenger RNA
m6A: N6-methyladenosine
m1A: N1-methyladenosine
DFSCs: Dental follicle stem cells
DLX5: Distal-less homeobox 5
RUNX2: Runt-related transcription factor 2
YAP: Yes-associated protein
DMP1: Dentin matrix protein-1
DMP2: Dentin matrix protein-2
Gro*α*: Growth related oncogene alpha
IL: Interleukin
TGF-*β*1: Transforming growth factor beta 1
HDAC2: Histone deacetylase 2
lncRNAs: Long noncoding RNAs
miRNAs: MicroRNAs
DNMT3b: DNA methyltransferase 3b
iPS cells: Induced pluripotent stem cells
KDM3B: Lysine demethylase 3B
KDM4B: Lysine demethylase 4B
KDM5A: Lysine demethylase 5A
siRNA: Small interfering RNA
TNF-*α*: Tumor necrosis factor-alpha
HDAC: Histone deacetylase 2
HDACi: Histone deacetylase inhibitor
EGFR: Epidermal growth factor receptor
JAK: Janus kinase
IC50: The half maximal inhibitory concentration
EZH2: Enhancer Of Zeste 2 Polycomb Repressive Complex 2 Subunit
KLF4: Krüppel-like factor 4.
